# Assessment of Job Stress of Clinical Pharmacists in Ho Chi Minh City, Vietnam: A Cross-Sectional Study

**DOI:** 10.3389/fpsyg.2021.635595

**Published:** 2021-04-28

**Authors:** Hai-Yen Nguyen-Thi, Minh-Thu Do-Tran, Thuy-Tram Nguyen-Ngoc, Dung Van Do, Luyen Dinh Pham, Nguyen Dang Tu Le

**Affiliations:** ^1^Faculty of Pharmacy, University of Medicine and Pharmacy at Ho Chi Minh City, Ho Chi Minh City, Vietnam; ^2^Ho Chi Minh City Department of Health, Ho Chi Minh City, Vietnam

**Keywords:** clinical pharmacists, job stress, healthcare profession stress inventory, Ho Chi Minh City, Vietnam

## Abstract

**Objectives:** The official implementation of clinical pharmacy in Vietnam has arrived relatively late, resulting in various stressors. This study aims to evaluate job stress level and suggest viable solutions.

**Methods:** A cross-sectional study was conducted on clinical pharmacists (CPs) in 128 hospitals in Ho Chi Minh City (HCMC). Job stress questions were derived from the Healthcare Profession Stress Inventory (HPSI).

**Results:** A total of 197 CPs participated, giving a response rate of 82.4%. Participants were found to have moderate job stress with an overall mean stress score of 1.5 (0.4) and stress rate of 52.8%. The sample size was statistically adequate and the HPSI was valid and reliable. Patient care responsibility was the main stressor, especially in public hospitals, followed by job conflicts. Lack of experience, low income, and inability to participate in clinical ward rounds caused significant stress to CPs regarding job recognition and job uncertainty. More practice-oriented training programs in bachelor curricula and clinical practice should be applied to help CPs gain more experience, self-confidence, and diminish job stress.

**Conclusion:** CPs in HCMC have moderate stress. More practice-oriented training programs should be prioritized to lessen stress for CPs.

## Introduction

Clinical pharmacy is a health science discipline in which pharmacists provide a patient-oriented service that optimizes medication therapy and promotes health, and disease prevention [American College of Clinical Pharmacy (ACCP), 2020a]. A diverse workload and the expectations of patients have heightened the level of working demands and apparent stress among the employees of hospitals. Stress can manifest itself through decreasing job satisfaction and increasing intentions to leave the organization (Gaither et al., 2008). A study in the US found that hospital pharmacists with the highest scores were least likely to choose their next role in the same profession (Wolfgang et al., 1988). Many pharmacists are suffering from stress at work due to various reasons related to their work environment, patients, or/and physicians (Olson and Lawson, 1996; Young, 1996; Lapane and Hughes, 2004; Sporrong et al., 2005; Schommer et al., 2006; Bryant et al., 2009; McCann et al., 2009; Yeh et al., 2010). Many studies have suggested that interpersonal conflict is one of the major reasons for pharmacists choosing to leave the profession and is one of the major predictors of stress in the workplace (Resnik et al., 2000; Kälvemark et al., 2004; Austin et al., 2010). As pharmacists struggle to establish relationships with physicians, having to work hard in order to build trust and a collaborative working relationship may contribute to pharmacists' stress in their workplace (Smith et al., 2002; Hughes and McCann, 2003; Kheir and Ali, 2008; Tahaineh et al., 2009; Snyder et al., 2010).

In Vietnam, clinical pharmacy was first introduced in the 1990s. However, it was only in 2012 that the Vietnamese Ministry of Health (MOH) imposed the first regulation, determining the role and tasks of a clinical pharmacist *(Circular No. 31/2012/TT-BYT)* (Ministry of Health in Vietnam, 2012). Recently, in 2020, with an aim of developing clinical pharmacy activities, the government of Vietnam issued a regulation on organizing and operating clinical pharmacy in medical facilities in Vietnam *(Decree No 131/2020/NÃ-CP)* (Government of Vietnam, 2020). Consequently, the late implementation of clinical pharmacy into healthcare practice has resulted in various barriers and limitations for CPs in Vietnam (Ho Chi Minh City Department of Health, 2020a,b; Nguyen-Thi et al., 2020).

Ho Chi Minh City (HCMC), located in the south of Vietnam, is one of the biggest healthcare centers in Vietnam with a huge number of inpatient and outpatient visits (Ho Chi Minh City Department of Health, 2020c). As a pioneer in implementing clinical pharmacy practice, HCMC has been confronted with various difficulties (Nguyen-Thi et al., 2020). In detail, a study conducted in 2019 showed that most hospitals in HCMC did not meet the standard of quality and quantity of clinical workforce according to MOH (Nguyen-Thi et al., 2020). Moreover, most clinical pharmacists (CPs) have to concurrently handle numerous traditional non-clinical tasks such as drug dispensing, compounding, and administrative tasks besides clinical pharmacy (Nguyen-Thi et al., 2020). All these reasons might raise job stress among CPs which further reduces job satisfaction, increases intentions to leave their jobs, and indirectly harms patients. Therefore, this study was carried out to evaluate the job stress level of CPs in hospitals in HCMC and suggest possible solutions to diminish stressors for CPs based on findings.

## Materials and Methods

### Study Design

This study was conducted in 128 hospitals in HCMC. These included 67 public hospitals and 61 private hospitals. Among the 67 public hospitals, the number of hospitals under direct administration of ministers, of the HCMC Department of Health, and of the District-level People's Committee were 12, 32, and 23, respectively.

This study was designed as a cross-sectional study. Job stress domain was based on a pre-validated questionnaire adopted from the Health Professions Stress Inventory (HPSI) (Wolfgang, 1988). The questionnaire was piloted on a convenience sample of 10.0% (*n* = 20) of the target sample for the purpose of Cronbach coefficient alpha test efficiency to assess the reliability of the questionnaire questions. The result was 0.883, which is satisfactory considering that 0.700 is the cut-off value for being acceptable (Santos, 1999). After piloting, some adjustments to wording based on pilot respondents' feedback and elimination of two inappropriate questions based on Item Total Correlation were made. The resulting data from the pilot test were excluded from the final analysis.

The questionnaire included three domains: (1) demographics, (2) job stress, and (3) free-response. The first domain included 13 questions about demographics and professional features. Questions of gender, marital status, professional degree, position, and number of tasks concurrently handled were multiple choice questions, and data of these were collected as categorical variables. The questions for the other demographics were free-response questions and those data were obtained as continuous variables. Domains of job stress were based on HPSI adjusted after pilot. The adjusted version consisted of 28 questions about job-related stress situations categorized into four subdomains with seven questions per subdomain: (i) professional recognition, (ii) patient care responsibility, (iii) job conflicts, and (iv) professional uncertainty (Wolfgang, 1988; Gupchup and Wolfgang, 1994). Participants were asked to rate how often they feel stressed with each situation on a 5-point Likert scale (0 = never and 4 = always). The final domain consisted of five free-response questions about reasons to stay and job-related stress factors. The full original questionnaire used in this study is shown in Supplementary File 1.

### Data Collection

Self-administered questionnaires were distributed to all CPs working in hospitals in HCMC through an online tool named Zoho from May to June 2020. Two separate survey links (link A and link B) were used in the data collection process. ***Link A*** comprised of two questions for heads/deputy heads of the Department of Pharmacy (HDPs) to report the number of CPs currently working in their hospitals and to check whether all CPs of each hospital had completed the questions at the end of the survey. ***Link B*** comprised of questionnaires for CPs. The data collection process consisted of three steps:

***Step 1***. Two separate surveys were emailed to all HDPs of hospitals in HCMC.
***Step 2***. HDPs of each hospital reported the number of CPs by filling in link A and then forwarding link B to CPs for their response.
***Step 3***. To ensure a good response rate, a reminder e-mail was sent to all HDPs 2 weeks afterwards.

### Statistical Analysis

SPSS version 20.0 was used for the analysis of the data. In the job stress domain, results were presented as mean (standard deviation), median (Q1-Q3), and number (percentage) of stressful answers. To determine the significant difference between means of two groups, *t*-test was applied. To be more specific, we used *t*-test to determine the significant difference between the means of two subgroups in gender (male, female), age (≤ 30, >30 years old), marital status (single, married), position (managers, employees), years of experience (≤10, >10 years), academic degree (B. Pharm, Post-graduates), monthly income (≤433 USD, >433 USD), on clinical ward rounds (yes, no), tasks concurrently handled (none, yes), and hospital settings (public, private). ANOVA was applied to determine the significant difference among three of the above subgroups in hospital types. The original publication only reported averages of points obtained by healthcare professionals and not proposed classification or cut points. However, based on several studies, the extent of stress was categorized into “no stress” (0.0–0.5), “mild stress” (> 0.5–1.5), “moderate stress” (> 1.5–2.5), “high stress” (> 2.5–3.5), and “extremely high stress” (> 3.5–4.0). The different degrees of stress were then grouped into “not stressed” (0.0–2.5) and “stressed” (> 2.5–4.0) (Smith et al., 2000; Nguyen, 2012; Munir et al., 2017; Palacios, 2020). The Chi square test was used to explore the relationship between demographics and being “stressed” or not. To determine factors associated with stress, multiple linear regression and binary logistic regression were used. The enter method was applied for both regressions. In multiple linear regression, the dependent variable was overall mean stress score and quantitative variables including age, years of experience, and monthly income were added as independent variables. In binary logistic regression, the dependent variable was feeling “stressed” or not and demographics; professional features were added as an independent variable with a cut-off value of 0.5. The odds ratio with a 95% confidence interval was calculated to measure the effect of independent variables on the likelihood of being “stressed.” Free-response answers were collected and coded into groups based on the main idea of each answer, then the frequency of answers of each group was calculated. A significance level of *p* < 0.05 was used, where the test was relevant.

## Results

Out of the 128 HDPs corresponding with 128 hospitals approached, 90 HDPs responded with the number of CPs and forwarded the survey link to all CPs at their hospital. The number of CPs reported from these 90 hospitals were 239. At the end of the study, it was determined that 197 out of 239 CPs participated, giving a response rate of 82.4%. The number of HDPs who responded and that of questionnaires returned by CPs in each type of hospital are summarized in Supplementary File 2.

### Demographics and Professional Features

**Figure 1:**
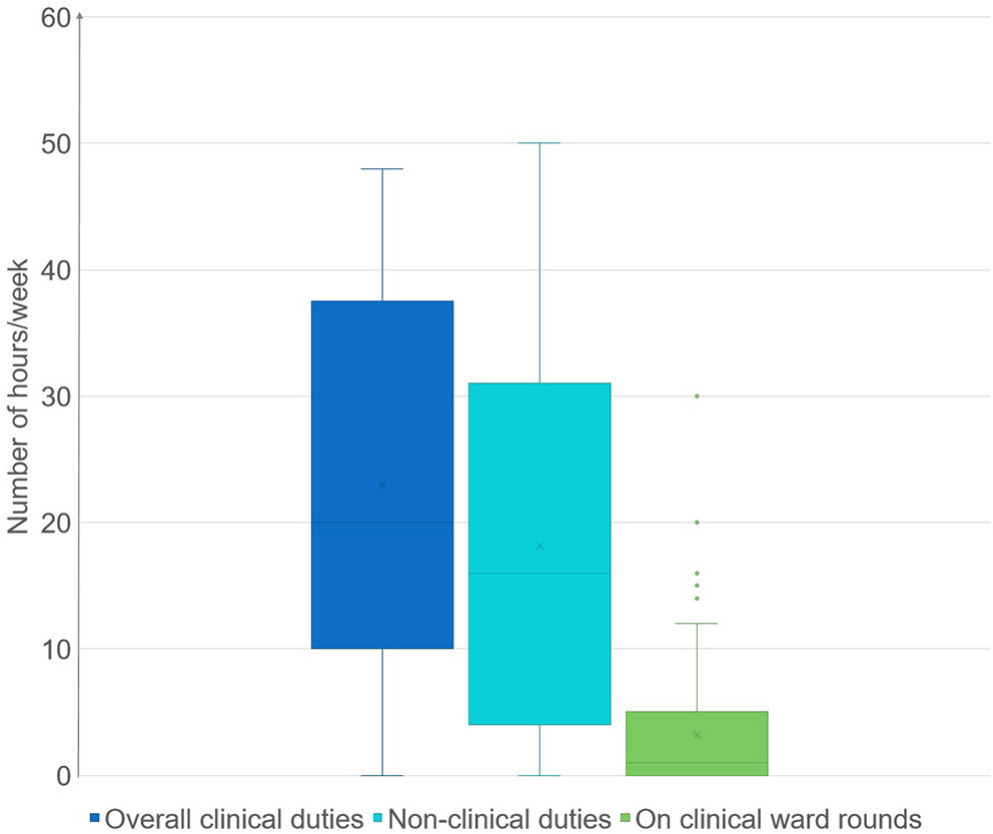
Time clinical pharmacists spent on each duty.

Table 1 depicts the demographics and professional features of the respondents. There were 150 (76.1%) females, and the average age was 32.3 (7.7). The average years of experience was 2.5 (3.0). Most respondents (83.8%) were working in public hospitals. Among those, 118 participants (59.9%) were working in public hospitals under direct administration of the HCMC Department of Health and 47 CPs (23.9%) were currently working in public hospitals under direct administration of the District-level People's Committee. Most (80.0%) had an income level lower than the average monthly income of inhabitants in HCMC. In terms of work-related characteristics, only about 30% of respondents were full-time CPs, while the remainder had to concurrently handle various tasks. The percentage of CPs who participated in clinical ward rounds with the clinical care team was relatively low (52.8%). The average hours that CPs spent on clinical pharmacy activities per week approximated the figure for other conventional pharmacy practice tasks, which were 23.17 (14.11) hours/week and 22.92 (12.44) hours/week, respectively. Nevertheless, the time spent on ward rounds was only a quarter as much as the total amount of time spent on clinical pharmacy activities. Details of time spent on each duty are presented in Figure 1.

**Table 1:** Demographics and profession characteristics.

**Category**	**Number (%)**
**Gender**	
Male	46 (23.4)
Female	150 (76.1)
Other	1 (0.5)
**Age**	
≤30	97 (49.0)
31–40	71 (36.0)
41–50	21 (11.0)
> 50	8 (4.0)
Mean (SD)^a^	32.3 (7.7)
Range^b^	24–60
**Marital status**	
Single	104 (52.3)
Married	93 (47.2)
Other	1 (0.5)
**Years of experience**	
≤5	177 (89.8)
6–10	16 (8.1)
>10	4 (2.1)
Mean (SD)^a^	2.5 (3.0)
Range^b^	0–25
**Hospital settings**	
Public	165 (83.8)
Public under HCMC DOH^d^	118 (59.9)
Public district-level^e^	47 (23.9)
Private	32 (16.2)
**Position**	
Head of Pharm. Dept	11 (5.6)
Deputy Head of Pharm. Dept	14 (7.1)
Official employee	118 (59.9)
Short-term employee	46 (23.4)
On-probation employee	8 (4.0)
**Degree**	
Bachelor	144 (73.1)
MSc	27 (13.7)
PhD	1 (0.5)
1st degree specialist	20 (10.2)
2nd degree specialist	5 (2.5)
Income per month
≤450 USD^c^	157 (80.0)
> 450 USD^c^	40 (20.0)
**Number of concurrent tasks**	
0	58 (29.4)
2	93 (47.2)
≥3	46 (23.4)
**On-ward participation**	
Yes	104 (52.8)
No	93 (47.2)

*Data are presented as number (percentage) if not stated, otherwise:*

a *Mean (standard deviation)*.

b *Range (minimum–maximum)*.

c *Average income of Ho Chi Minh City inhabitants*.

d *Public hospitals under direct administration of the Ho Chi Minh City Department of Health*.

e *Public hospitals under direct administration of the District-level People's Committee*.

### Job-Related Stress

**Figure 2:**
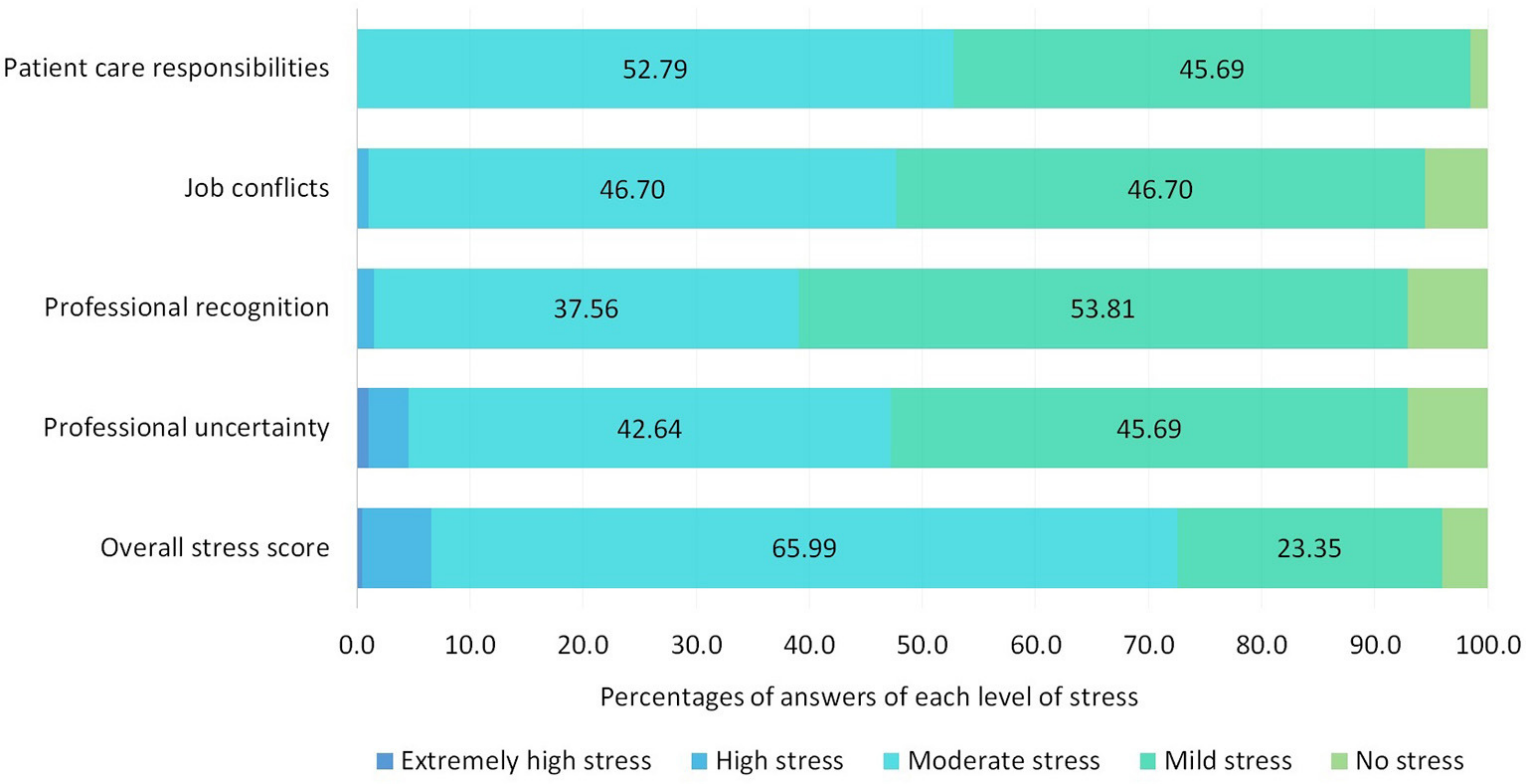
Number of answers of each level of stress for each subset and overall domain.

Overall, the surveyed CPs reported a moderate level of stress with an overall mean score of 1.5 (0.4) and a stress rate of 52.8%. Among four main factors of stressors, patient care responsibility had the highest mean stress score of 1.8 (0.6), which caused stress to 72.6% of CPs, followed by job conflicts and professional uncertainty with percentages of 47.2 and 46.7%, respectively. Professional recognition caused lower stress compared to other factors (39.1%). Details of stress score are shown in Table 2 and Figure 2. Among 29 situations, the 10 most stressful ones are shown in Table 3. Among the 10 highest stressful situations, five were related to patient care responsibilities, followed by two of job conflicts, two of professional recognition, and one of professional uncertainty. Full results of stress felt for each situation are shown in Supplementary File 3.

**Table 2:** Average stress score of each subdomain and overall job stress domain.

**Category**	**Cronbach's alpha**	**Rank order**	**Mean (SD)**	**Median (Q1–Q3)**	**Number (%)^a^**
Patient care responsibility	0.851	1	1.8 (0.6)	1.9 (1.4–2.0)	143 (72.6)
Job conflicts	0.847	2	1.5 (0.7)	1.4 (1.1–1.9)	93 (47.2)
Professional uncertainty	0.750	4	1.4 (0.5)	1.4 (1.1–1.8)	92 (46.7)
Professional recognition	0.862	3	1.4 (0.6)	1.3 (1.0–1.8)	77 (39.1)
Overall stress score	0.889		1.5 (0.4)	1.5 (1.3–1.8)	104 (52.8)

a *Number (percentage) of stressful answers*.

*Stressful answers are defined by a score of more than 1.5*.

**Table 3:** Rank orders of 10 most stressful situations.

**Category**	**Rank order**	**Mean (SD)**	**Number (%)^a^**
Trying to meet society's expectations for high quality medical care	1	2.4 (1.0)	169 (85.8)
Not having enough staff to provide necessary services adequately	2	2.0 (0.9)	149 (75.6)
Dealing with “difficult” patients	3	1.8 (0.8)	147 (74.6)
Fearing that a mistake will be made in the treatment of a patient	4	1.9 (0.9)	145 (73.6)
Caring for terminally ill patients	5	1.8 (0.8)	141 (71.6)
Having so much work to do that everything cannot be done well	6	1.8 (0.8)	138 (70.1)
Feeling ultimately responsible for patient outcomes	7	1.7 (0.9)	133 (67.5)
Feeling that opportunities for advancement on the job are poor	8	1.8 (0.8)	132 (67.0)
Caring for the emotional needs of patients	9	1.7 (0.8)	132 (67.0)
Feeling that you are inadequately paid as a health professional	10	1.8 (0.9)	117 (59.4)
Being uncertain about what to tell a patient or their family about the patient's condition and/or treatment	10	1.7 (0.8)	117 (59.4)

a *Number (percentage) of stressful answers*.

*Stressful answers are defined by a score of more than 1.5*.

The association between demographics, work-related characteristics, and job stress is described in Table 4. The mean stress score of each subgroup of gender (male, female), position (manager, employee), tasks concurrently handled (none, yes), and hospital settings (public, private) were significantly different. Furthermore, in comparison among public hospitals under direct administration of the HCMC Department of Health, public hospitals under direct administration of the District-level People's Committee, and private hospitals, the difference in overall stress score was not significant (*p* > 0.05) in the ANOVA test. CPs under 30 years old felt more stressed due to professional uncertainty (*p* = 0.001), and CPs who had < 5 years of experience felt more stressed than their senior colleagues due to professional recognition (*p* = 0.042). People who work in public hospitals felt more stressed by patient care responsibility compared to those in private hospitals (*p* = 0.015). Furthermore, CPs who had a monthly income <450 USD felt more stressed due to professional recognition (*p* = 0.004) and professional uncertainty (*p* = 0.039) than those who earned more. The correlation between mean stress score and age, years of experience and monthly income was not statistically significant (*p* > 0.05). In multiple linear regression, the *R* square was 0.026, and the independent variables were not statistically significantly predictors with *F*_(4,191)_ = 1.273, *p* > 0.05. Therefore, the multiple linear regression was not a good fit of the data.

**Table 4:** Job stress in relation to demographics and professional features.

**Category**	**Mean (SD)^a^**	**Spearman correlation^c^**	**Linear regression coefficient^d^**	**Not stressed**	**Stressed**	***p*****-value^e^**	**OR (95%CI)^g^**
**Gender^f^**							
Male	**1.64 (0.37)**			18	28	0.23	1.00
Female	**1.46 (0.42)**			74	76		0.54 (0.26–1.12)
**Age**		−0.022	−0.09				
≤30	1.53 (0.40)			45	52	0.82	1.00
>30	1.48 (0.42)			48	52		0.98 (0.93–1.03)
**Marital status^f^**							
Single	1.50 (0.38)			52	50	0.34	1.00
Married	1.50 (0.45)			40	53		1.58 (0.82–3.50)
**Position**							
Managers	**1.33 (0.55)**			16	9	0.09	**1.00**
Employees	**1.53 (0.39)**			77	95		**3.24 (1.05–10.45)**
**Years of experience**	0.107	0.013	0.013			
< =10	1.50 (0.42)			84	93	0.84	1.00
>10	1.62 (0.20)			9	11		1.05 (0.94–1.17)
**Academic degree**							
B. Pharm	1.51 (0.40)			69	75	0.74	1.00
Postgraduate	1.49 (0.45)			24	29		1.07 (0.48–2.39)
**Monthly income**	−0.004	0.001	0.001			
≤450 USD	1.53 (0.40)			75	82	0.75	1.00
>450 USD	1.40 (0.46)			18	22		1.02 (0.93–1.13)
**On clinical ward rounds**							
Yes	1.46 (0.40)			**37**	**56**	**0.04**	1.00
No	1.55 (0.42)			**56**	**48**		1.02 (0.95–1.09)
**Tasks concurrently handled**							
None	**1.40 (0.38)**			**35**	**23**	**0.04**	**1.00**
Yes	**1.55 (0.42)**			**58**	**81**		**2.75 (1.35–5.60)**
**Hospital settings**							
Public	**1.53 (0.39)**			75	90	0.26	1.00
Private	**1.38 (0.48)**			18	14		1.02 (0.41–2.56)
**Hospital types^b^**							
Public under HCMC DOH^h^	1.55 (0.40)			52	66	0.46	1.00
Public district-level^i^	1.48 (0.40)			23	24		0.70 (0.34–1.46)
Private	1.38 (0.48)			18	14		0.86 (0.35–2.16)

a *Mean stress scores are compared between subgroups by using t-test*.

b *Mean stress scores were compared among public hospitals under direct administration of the HCMC Department of Health, public hospitals under direct administration of the District-level People's Committee, and private hospitals using ANOVA*.

c *Spearman correlation with overall mean stress score*.

d *Coefficient in multiple linear regression*.

e *p-value of Chi square test. “Stressed” answers are defined by a score of more than 1.5*.

f *Sum does not equal 197 as some groups were excluded to satisfy the Chi-square or Fisher test condition*.

g *Odds ratio with 95% confidence interval in binary logistic regression*.

h *Public hospitals under direct administration of the Ho Chi Minh City Department of Health*.

i *Public hospitals under direct administration of the District-level People's Committee*.

*Bold data indicates a statistically significant difference with a p < 0.05*.

The characteristics noted to have statistically significant correlations with overall job stress were on-ward participation (*p* = 0.04) and covering concurrent tasks (*p* = 0.04). Regarding on-ward participation, CPs who did not participate in clinical ward rounds felt more stressed than their counterparts due to professional recognition (*p* = 0.006) and professional uncertainty (*p* = 0.002). Considering the number of concurrent tasks, CPs who had to cover tasks felt more stressed due to overall job stress score (*p* = 0.022) and job conflicts (*p* = 0.000) than their counterparts. Results of the Chi square test are shown in Table 4. The binary logistic regression model was statistically significant (χ^2^ = 18.131, df = 10, *p* < 0.05). The results indicated that only position (OR = 3.24, 95% CI: 1.05–10.45) and covering concurrent tasks (OR = 2.75, 95% CI: 1.35–5.60) were the predictors of feeling “stressed.” Employees were 3.24 times more likely to feel “stressed” than managers and CPs who cover concurrent tasks were 2.75 times more likely to feel “stressed” than their counterparts. Hence, being an employee (rather than a manager) and covering tasks were associated with the likelihood of feeling “stressed.” The odds ratio results of the binary logistic regression are shown in Table 4.

### Free-Response Domain

Regarding satisfactory factors that keep CPs working in current hospitals, friendly working environment was highly mentioned by 97 CPs (49.2%), followed by worker characteristics including willingness to learn and gain experience and passion for the profession with 79 (40.1%) and 75 (38.1%), respectively. Regarding stressors, working conditions comprising the lack of a stringent theoretical framework, low income, work overload, and shortage of staff were repeatedly noted by 12.2–25.4% of CPs. Worker characteristics including lack of collaboration with other healthcare professionals, especially physicians, and lack of acknowledgment and experience was mentioned by 26.4 and 18.3%, respectively.

## Discussion

This is the first study to evaluate the job stress level of CPs in Vietnam. The overall response rate of 82.4% was acceptable. Participants were mostly female (76.1%) and single (52.3%). Most of the participants were young and beginner CPs with 85% < 40 years old and 89.8% with < 5 years of experience. This result is comparable to the study on clinical pharmacy workforce in HCMC in 2018 where the median years of experience was 2 (Q1-Q3: 2-5) (Nguyen-Thi et al., 2020). The low working seniority of CPs might be due to the late implementation of clinical pharmacy (Vietnamese Ministry of Health, 2012). The academic requirement for a CP in Vietnam is less stringent than other countries like the US, where a Doctorate of Pharmacy is compulsory [Vietnamese Ministry of Health, 2012; American College of Clinical Pharmacy (ACCP), 2020b]. As a result, most CPs have a Bachelor's degree in Pharmacy (73.1%). This result is comparable to the study on a workforce of CPs in HCMC in 2018 (Nguyen-Thi et al., 2020).

### Overall Stress Score

Clinical pharmacists felt moderate stress with a mean score of 1.5 (0.4) and 104 (52.8%) CPs felt stressed with their current job. This study has a similar result to two studies on hospital pharmacists where more than 68.0% experienced job stress (Wolfgang et al., 1988; Mott et al., 2004). In Vietnam, this study had a similar result with a multisite study in Hanoi with 48.6% of healthcare professionals (HCPs) reporting stress (Nguyen and Doan, 2020). However, this proportion is relatively lower than a study on psychological HCPs in 2015 in Vietnam (Lai et al., 2015). This higher percentage can be explained by high frequency of exposure with psychological patients in this sector. The result of a study conducted in Hai Phong in 2011 had a lower stress portion with 6.39%, which might be due to the lower number of patient visits compared to HCMC (Pham and Hoang, 2011). This study found that male CPs had significantly higher stress levels than their counterparts in overall stress score and all stressors except for professional recognition (*p* < 0.05). Moreover, CPs working in public hospitals experienced significantly more stress at work than those in private hospitals. However, in comparison, among public hospitals under direct administration of the HCMC Department of Health, public hospitals under direct administration of the District-level People's Committee, and private hospitals, the difference in overall stress score was not significant (*p*>0.05).

### Patient Care Responsibility

Patient care responsibility was the main stressor for CPs with the highest mean stress score of 1.8 (0.6), ranking number 5 in the top 10 stressful situations. Most CPs (72.6%) felt stress in providing patient-oriented care. The traditional role of pharmacists in hospitals in Vietnam revolves around drug dispensing, compounding, and administrative tasks. New stressors include face-to-face interactions with patients in the clinical role of CPs. In detail, the most stressful situations for CPs in patient care responsibility was the effort to meet society's expectations, which caused stress for 85.8% of CPs and had a mean stress score of 2.4 (1.0), which means more than half of CPs often to very often felt stressed with this situation. Besides, confronted with “difficult” and terminally ill patients, feeling ultimately responsible for patient outcomes, and caring for the emotional needs of patients also put high pressure on more than two-thirds of CPs (67.0–74.6%). A national study in the US also supports this theory as the sources of stress related to patient care for nurses were significantly higher than those of CPs, and the increasing need of patient care in the clinical role of CPs would put more pressure on them (Wolfgang et al., 1988).

Furthermore, this study also found that CPs who were working in public hospitals had significantly higher stress scores in patient care responsibility than those in private hospitals (*p* = 0.015). This study also noted that the public hospital group had a higher mean stress score than their counterparts. A study in Jordan also had a similar result, in which patient care responsibility was significantly associated with the type of pharmacy practice settings (*p* = 0.043), and public hospitals had a higher stress score than others (Al Khalidi and Wazaify, 2013). This can be explained by the high intensity of workload in public hospitals compared to private ones, which does not permit CPs to routinely follow-up with prescribers and results in making CPs feel uncertain in consulting patients. The expectation for the new clinical role of CPs from society, patients, and other HCPs has heightened the level of stress for them. Long-time stress in professionals who work continuously with people, like in clinical pharmacy, can be emotionally exhausting and raise the risk of burnout (Maslach and Jackson, 1981; Schaufeli and Enzmann, 1998; Eslami et al., 2016). However, a study measuring moral distress in Sweden noted that CPs reported more tolerance toward moral dilemmas than medical staff (Sporrong et al., 2006). This is a positive signal that CPs would be able to suffer less job stress related to patient care responsibility.

### Job Conflicts

Job conflicts is the second biggest stressor for CPs which caused stress to 47.2% of CPs and was ranked number 2 in the top 10 stressful situations. Shortage of staff along with work overload promote enormous stress levels in a vast proportion of CPs, 75.6 and 70.1%, respectively. Besides, in the free-response questions, a majority of CPs indicated that shortage of staff and work overload, which force them to multi-task and work longer hours, made them feel very stressed. These factors are also mentioned in various studies on big stressors that affect CPs' abilities to perform their duties (Wolfgang et al., 1988; Lapane and Hughes, 2006; Rothmann and Malan, 2007; McCann et al., 2009; Al Khalidi and Wazaify, 2013; Amal, 2015). In addition, this study also found that 70.6% of CPs had to cover concurrent tasks and those who had to cover tasks had significantly higher stress scores for overall stress score and job conflicts than their counterparts (*p* < 0.05). CPs having to handle concurrent tasks were 2.75 times more likely to feel “stressed.” As clinical pharmacy is still new in Vietnam, the number of available staff is relatively low (Vietnamese Ministry of Health, 2012). A study in HCMC in 2018 found that there is an imbalance in healthcare staffing with a ratio of nurses/physicians/CPs of 92.4/42.1/1.0 (Nguyen-Thi et al., 2020). Consequently, CPs have to cover various tasks including clinical and non-clinical ones (Nguyen-Thi et al., 2020). Other conflicts with coworkers, supervisors, or administrators in the HPSI domain were not stressful. However, in the free-response domain, lack of collaboration and respect from HCPs, especially physicians made many CPs feel stressed. In detail, some CPs indicated that the misperception toward the clinical role of CPs led to incorporation with HCPs, especially physicians. This result is similar with a study in Jordan, where the implementation of clinical pharmacy is relatively new like in Vietnam. The study in Jordan found that job conflicts with other HCPs, especially with physicians, occur daily in hospitals and cause a significant amount of stress for CPs, and physicians were less likely to accept newer clinical services delivered through pharmacists (Tahaineh et al., 2009; Al Khalidi and Wazaify, 2013).

### Professional Recognition and Professional Uncertainty

Professional recognition and professional uncertainty had a lower impact on CPs' stress with stress scores of 1.4 (0.5) and 1.4 (0.6), respectively. Lack of opportunities for advancement and inadequate salary were two main stressors in professional recognition which caused stress to 67.0 and 59.4% of CPs, respectively. Besides, the CPs in the free-response questions mentioned that low income was a very stressful factor related to their jobs. These stressors were also noted in other studies (Wolfgang et al., 1988; Rothmann and Malan, 2007; Al Khalidi and Wazaify, 2013; Amal, 2015). Regarding position, the employees group had a significantly higher mean stress score than the managers group with 1.53 (0.39) and 1.33 (0.55), respectively. In addition, in the binary logistic regression, employees were 3.24 times more likely to feel “stressed” than managers. Regarding salary, a majority of 80.0% of CPs had an income lower than 450 USD—the average income of inhabitants in HCMC. A study of clinical pharmacy workforce in 2018 noted that the number of CPs who had an income in the bottom half (<520 USD/month) notably outweighed that in the top half of the list (Nguyen-Thi et al., 2020). This result is comparable to previously reported statistics in Vietnam, where CPs (≤2 years of experience) had an average income below 430 USD, equivalently in 2020 (Salary Explorer, 2019). However, this level of income was relatively low compared to other HCPs (First Alliances, 2019). Furthermore, this study also found that CPs who had income lower than 450 USD felt more stressed due to professional recognition and professional uncertainty than those who had a higher income (*p* < 0.05). Fear of making a mistake during treatment and uncertainty in consulting patients were the two highest stressors in professional uncertainty which caused stress to 73.6 and 59.4% of CPs, respectively. This can be explained by inadequate patient condition information (39.1%) and lack of experience in doing the clinical task of consulting the patient/family members of patient. This led CPs to feel uncertain about what to tell the patient or how to counsel him/her, and failure to provide proper counseling can result in ineffective medications that might adversely affect the health and safety of patients (Bond et al., 2000; Resnik et al., 2000).

Furthermore, regarding age and work experience, most of participants were young and beginner CPs with 85% < 40 years old and 89.8% had < 5 years of experience. This study found that CPs younger than 30 years old experienced more stress due to professional uncertainty and junior CPs who had < 5 years of experience felt more stressed due to professional recognition. It is a reasonable finding due to their lack of experience. Consequently, feeling inadequate in clinical-related issues and therapeutic knowledge will affect CPs' self-confidence, and thus increase stress levels. Therefore, Bryant et al. suggested providing pharmacists with training programs in an attempt to overcome insufficient clinical knowledge and skills, which would help boost their self-confidence (Bryant et al., 2009).

This study also found that nearly half of CPs (52.8%) participated in clinical ward rounds and those who participated had less stress regarding professional recognition and professional uncertainty than those who did not (*p* < 0.05). Therefore, participating in clinical ward rounds also plays a vital role in making CPs feel equal to HCPs in a clinical care team. Furthermore, in the free-response questions, a majority stated that passion for the profession and willingness to learn and gain experience helped them stay in their jobs. However, lack of knowledge and low collaboration with other HCPs, especially with physicians make them stressed. The incorporation with other HCPs can be explained by the mild theoretical framework of CPs which frustrate HCPs when trying to identify and differentiate CPs' responsibilities. Recently, a regulation on organizing and operating activities in clinical pharmacy in healthcare facilities was issued and came into effect on January 1, 2021 (Government of Vietnam, 2020). This decree not only defines the role and tasks of CPs and targets clinical pharmacy activities and workforce but also regulates the long-term plans for each kind of medical facility. This more precise decree could help clinical pharmacy improve in activities and quality. Besides, when CPs struggle to establish relationships with physicians, they sometimes have to work hard in order to build trust and a collaborative working relationship, which may contribute to pharmacists' stress in their workplace (Smith et al., 2002; Hughes and McCann, 2003; Kheir and Ali, 2008; Tahaineh et al., 2009; Snyder et al., 2010). Thus, more practice in consulting patients and cooperating with other HCPs in a clinical care team in clinical practice, continuous pharmacy education (CPE) to update drug information and clinical knowledge along with a more practice-oriented teaching program in Bachelor curricula like interprofessional education (IPE) should be done to help CPs gain more experience and self-confidence.

### Limitations

This study could not capture all CPs at HCMC as it was conducted during the outbreak of the COVID-19 pandemic and some CPs were too busy to complete the survey. Second, the study was conducted before the implementation of the new government regulation on organizing and operating clinical pharmacy in medical facilities in Vietnam (Decree 131/2020/NÃ-CP). Therefore, this study cannot evaluate the impact of this new regulation on job-related stress factors on CPs.

## Conclusions

The surveyed CPs had a moderate stress rate. Patient care responsibility was the highest score stressor. Further education and training for both CPs and clinical pharmacy students should be prioritized to help lessen the stressors of CPs to prevent the loss of a greatly educated and valuable workforce.

## Data Availability Statement

The raw data supporting the conclusions of this article will be made available by the authors, without undue reservation.

## Ethics Statement

Ethical review and approval was not required for the study on human participants in accordance with the local legislation and institutional requirements. The patients/participants provided their written informed consent to participate in this study. Written informed consent was obtained from the individual(s) for the publication of any potentially identifiable images or data included in this article.

## Author Contributions

H-YN-T and NL designed the study and acquired the data from the literature. M-TD-T, T-TN-N, H-YN-T, and NL ran the analysis. LP and DD provided expert advice and further contributed to the acquisition of data. M-TD-T, H-YN-T, and NT drafted the manuscript which was reviewed and revised by all authors. All authors have contributed substantially to the study, checked the assumptions ranges, interpreted the results, and approved the final submitted version of the manuscript.

## Conflict of Interest

The authors declare that the research was conducted in the absence of any commercial or financial relationships that could be construed as a potential conflict of interest.

## Supplementary Material

Supplementary File 1 Original questionnaire.

Supplementary File 2 Summary of the number of HDPs who responded and that of questionnaires returned by CPs in each type of hospital.

Supplementary File 3 Full results of stress felt for each situation.
