# Supplementary material for: Assessment of Job Stress of Clinical Pharmacists in Ho Chi Minh City, Vietnam: A Cross-Sectional Study
Source: Front Psychol. 2021 Apr 28;12:635595. doi: 10.3389/fpsyg.2021.635595 (PMC8113390; doi:10.3389/fpsyg.2021.635595)
Supplement: Supplementary File 1 — Original questionnaire. [file Data_Sheet_1.docx]

**Supplementary File 1. Original questionnaire**

| University of Medicine and Pharmacy  at Ho Chi Minh City  **Pharmaceutical Management Department** | **SOCIALIST REPUBLIC OF VIETNAM**  Independence – Freedom – Happiness |
| --- | --- |
|  | \| *Survey ticket code*: **DSLS_CTCV_** \|  \|  \|  \| \| --- \| --- \| --- \| --- \| |

**SURVEY QUESTIONNAIRE**

**ASSESSMENT OF JOB STRESS OF CLINICAL PHARMACISTS
IN HO CHI MINH CITY, VIETNAM**

Dear Pharmacists,

Our research group of the Pharmaceutical Management Department (University of Medicine and Pharmacy at Ho Chi Minh City) in conjunction with the HCMC Department of Health are implementing a research on job stress of clinical pharmacists. In fact, clinical pharmacy is a relatively new field in Vietnam. Therefore, stress factors at work of clinical pharmacists have not been considered and measured properly. Therefore, the main objective of this study is to identify the most stressful factors at work of clinical pharmacists to make recommendations in the policy. We hope to receive the support of pharmacists and we hope that you can take a little valuable time to complete the survey below.

Your information will not be disclosed and only used for research purposes. Time estimated to complete this survey would be 15 minutes. We are looking forward for your participation.

Thanks & Best Regards!

**DOMAIN A. PERSONAL INFORMATION**

**Please complete the information below by ticking the box or by filling in the blanks:**

**1. Gender:** 🞏 1. Male 🞏 2. Female 🞏 3. Other

**2. Marital status:** 🞏 1. Single 🞏 2. Married 🞏 3. Other

**3. Year of birth:**

**4. Year of starting to participate in clinical pharmacy (at the hospital):**

**5. Name of hospital currently working in:**

**6. Department currently working in:**

**7. Professional degree:** 🞏 1. Bachelor 🞏 4. First degree specialist

🞏 2. MSc 🞏 5. Second degree specialist

🞏 3. PhD

**8. Professional position:** 🞏 1. Head of Pharm.Dept 🞏 4. Short-term employee

🞏 2. Deputy Head of Pharm.Dept 🞏 5. Other:

🞏 3. Official employee

**9. Number of tasks concurrently handling:**

🞏 1. One task only 🞏 2. Two tasks 🞏 3. From 3 tasks to above

**10. Number of hours for overall clinical duties:**

**11. Number of hours for non-clinical duties:**

**12. Number of hours participating in clinical ward rounds:**

**13. Monthly income (million vnd):**

**DOMAIN B. QUESTIONNAIRE**

**Please indicate how often you encounter the following problems by ticking the number that corresponds to the answer you think are most appropriate with 0=never, 1=almost never, 2=sometimes, 3=frequently, 4=always.**

| **Situations** | **0** | **1** | **2** | **3** | **4** |
| --- | --- | --- | --- | --- | --- |
| 1. Feeling that opportunities for advancement on the job are poor | 0 | 1 | 2 | 3 | 4 |
| 2. Feeling that you are inadequately paid as a health professional | 0 | 1 | 2 | 3 | 4 |
| 3. Not receiving adequate feedback on your job performance | 0 | 1 | 2 | 3 | 4 |
| 4. Not being allowed to participate in making decisions about your job | 0 | 1 | 2 | 3 | 4 |
| 5. Not receiving the respect or recognition that you deserve from the general public | 0 | 1 | 2 | 3 | 4 |
| 6. Not having opportunities to share feelings and experiences with colleagues | 0 | 1 | 2 | 3 | 4 |
| 7. Not being able to use your abilities to the fullest extent on the job | 0 | 1 | 2 | 3 | 4 |
| 8. Trying to meet society's expectations for high-quality medical care | 0 | 1 | 2 | 3 | 4 |
| 9. Dealing wirh "difficult" patients | 0 | 1 | 2 | 3 | 4 |
| 10. Caring for terminally ill patients | 0 | 1 | 2 | 3 | 4 |
| 11. Feeling ultimately responsible for patient outcomes | 0 | 1 | 2 | 3 | 4 |
| 12. Caring for the emotional needs of patients | 0 | 1 | 2 | 3 | 4 |
| 13. Keeping up with new developments in order to maintain professional competence | 0 | 1 | 2 | 3 | 4 |
| 14. Disagreeing with other health professionals concerning the treatment of a patient | 0 | 1 | 2 | 3 | 4 |
| 15. Not having enough staff to provide necessary services adequately | 0 | 1 | 2 | 3 | 4 |
| 16. Having so much work to do that everything cannot be done well | 0 | 1 | 2 | 3 | 4 |
| 17. Having job duties which conflict with family responsibilities | 0 | 1 | 2 | 3 | 4 |
| 18. Being interrupted by phone calls or people while performing job duties | 0 | 1 | 2 | 3 | 4 |
| 19. Supervising the performance of coworkers | 0 | 1 | 2 | 3 | 4 |
| 20. Experiencing conflicts with supervisors and/or administrators | 0 | 1 | 2 | 3 | 4 |
| 21. Experiencing conflicts with coworkers | 0 | 1 | 2 | 3 | 4 |
| 22. Fearing that a mistake will be made in the treatment of a patient | 0 | 1 | 2 | 3 | 4 |
| 23. Being uncertain about what to tell a patient or family about the patient's condition and/or treatment | 0 | 1 | 2 | 3 | 4 |
| 24. Possessing inadequate information regarding a patient's medical condition | 0 | 1 | 2 | 3 | 4 |
| 25. Allowing personal feelings/emotions to interfere with the care of patients | 0 | 1 | 2 | 3 | 4 |
| 26. Being inadequately prepared to meet the needs of patients | 0 | 1 | 2 | 3 | 4 |
| 27. Not knowing what type of job performance is expected | 0 | 1 | 2 | 3 | 4 |
| 28. Not being recognized or accepted as a true health professional by other health professionals | 0 | 1 | 2 | 3 | 4 |

**Please write your answer by filling in the blanks.**

**1. Can you indicate the reason that keeps you continue to work here?**

**2. What makes you feel satisfied with your current work?**

**3. What makes you feel NOT satisfied with your current work?**

**4. What else makes you feel stressed with your current work?**

**5. What changes could be made to help reduce your stress at work?**
