# Supplementary figures and images for: Assessment of Job Stress of Clinical Pharmacists in Ho Chi Minh City, Vietnam: A Cross-Sectional Study
Source: Front Psychol. 2021 Apr 28;12:635595. doi: 10.3389/fpsyg.2021.635595 (PMC8113390; doi:10.3389/fpsyg.2021.635595)

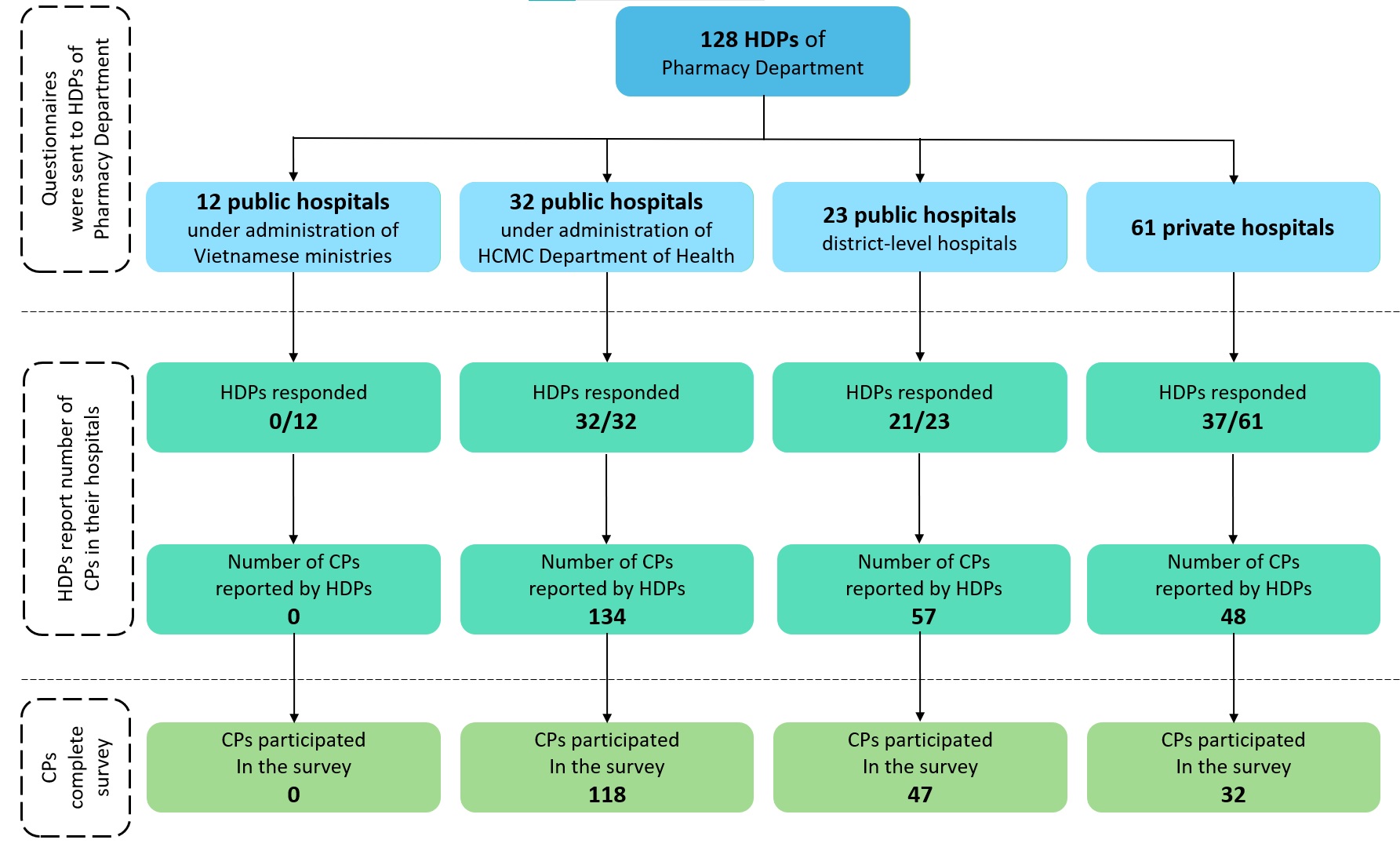

Supplement: Supplementary File 2 — Summary of the number of HDPs who responded and that of questionnaires returned by CPs in each type of hospital. [file Image_1.JPEG]
