# Supplementary material for: Assessment of Job Stress of Clinical Pharmacists in Ho Chi Minh City, Vietnam: A Cross-Sectional Study
Source: Front Psychol. 2021 Apr 28;12:635595. doi: 10.3389/fpsyg.2021.635595 (PMC8113390; doi:10.3389/fpsyg.2021.635595)
Supplement: Supplementary File 3 — Full results of stress felt for each situation. [file Table_1.docx]

**Supplementary File 3. Mean score (SD) and number (%) of stress of each situation**

| **Category** | **Mean (SD)** | **Number (%)** |
| --- | --- | --- |
| **Professional recognition** |  |  |
| Feeling that opportunities for advancement on the job are poor | 1.8 (0.8) | 132 (67.0) |
| Feeling that you are inadequately paid as a health professional | 1.8 (1.0) | 117 (59.4) |
| Not receiving adequate feedback on your job performance | 1.4 (0.7) | 79 (40.1) |
| Not being allowed to participate in making decisions about your job | 1.3 (0.7) | 76 (38.6) |
| Not receiving the respect or recognition that you deserve from the general public | 1.2 (0.7) | 61 (30.1) |
| Not having opportunities to share feelings and experiences with colleagues | 1.2 (0.7) | 58 (29.4) |
| Not being able to use your abilities to the fullest extent on the job | 1.0 (0.7) | 39 (19.8) |
| **Patient care responsibilities** |  |  |
| Trying to meet society's expectations for high-quality medical care | 2.4 (1.0) | 169 (85.8) |
| Dealing wirh "difficult" patients | 1.8 (0.8) | 147 (74.6) |
| Caring for terminally ill patients | 1.8 (0.8) | 141 (71.6) |
| Feeling ultimately responsible for patient outcomes | 1.7 (0.9) | 133 (67.5) |
| Caring for the emotional needs of patients | 1.7 (0.8) | 132 (67.0) |
| Keeping up with new developments in order to maintain professional competence | 1.4 (1.0) | 98 (49.7) |
| Disagreeing with other health professionals concerning the treatment of a patient | 1.5 (0.8) | 67 (34.0) |
| **Job conflicts** |  |  |
| Not having enough staff to provide necessary services adequately | 2.0 (0.9) | 149 (75.6) |
| Having so much work to do that everything cannot be done well | 1.8 (0.8) | 138 (70.1) |
| Having job duties which conflict with family responsibilities | 1.6 (0.9) | 112 (56.9) |
| Being interrupted by phone calls or people while performing job duties | 1.5 (0.9) | 112 (56.9) |
| Supervising the performance of coworkers | 1.5 (1.1) | 101 (51.3) |
| Experiencing conflicts with supervisors and/or administrators | 0.9 (0.8) | 41 (20.8) |
| Experiencing conflicts with coworkers | 0.9 (0.9) | 29 (14.7) |
| **Professional Uncertainty** |  |  |
| Fearing that a mistake will be made in the treatment of a patient | 1.9 (0.9) | 145 (73.6) |
| Being uncertain about what to tell a patient or family about the patient's condition and/or treatment | 1.7 (0.8) | 117 (59.4) |
| Possessing inadequate information regarding a patient's medical condition | 1.3 (0.7) | 77 (39.1) |
| Allowing personal feelings/emotions to interfere with the care of patients | 1.3 (0.8) | 74 (37.6) |
| Being inadequately prepared to meet the needs of patients | 1.3 (0.8) | 67 (34.0) |
| Not knowing what type of job performance is expected | 1.2 (0.7) | 55 (27.9) |
| Not being recognized or accepted as a true health professional by other health professionals | 1.3 (0.8) | 49 (24.9) |
